# Hepatoprotective Actions of Ascorbic Acid, Alpha Lipoic Acid and Silymarin or Their Combination Against Acetaminophen-Induced Hepatotoxicity in Rats

**DOI:** 10.3390/medicina55050181

**Published:** 2019-05-21

**Authors:** Anmar M. Abdulrazzaq, Mujtaba Badr, Omar Gammoh, Asad A. Abu Khalil, Bayan Y. Ghanim, Tawfiq M. Alhussainy, Nidal A. Qinna

**Affiliations:** 1 Department of Pharmacology and Biomedical Sciences, Faculty of Pharmacy and Medical Sciences, University of Petra, P.O. Box 961343, Amman 11196, Jordan; anmar.moaid@gmail.com (A.M.A.); talhussainy@yahoo.com (T.M.A.); 2 University of Petra Pharmaceutical Center (UPPC), University of Petra, P.O. Box 961343, Amman 11196, Jordan; mujtaba.badr@usask.ca (M.B.); aabukhalil@uop.edu.jo (A.A.A.K.); ghanimbayan1@gmail.com (B.Y.G.); 3 Department of Pharmacy, Faculty of Health Sciences, American University of Madaba, P.O. Box 2882, Madaba 11821, Jordan; o.gammouh@aum.edu.jo

**Keywords:** acetaminophen, ascorbic acid, silymarin, hepatoprotection, hepatotoxicity, hepatocytes, oxidative stress

## Abstract

*Background and objectives:* Ascorbic acid, alpha lipoic acid (ALA) and silymarin are well-known antioxidants that have hepatoprotective effects. This study aims to investigate the effects of these three compounds combined with attenuating drug-induced oxidative stress and cellular damage, taking acetaminophen (APAP)-induced toxicity in rats as a model both in vivo and in vitro. *Materials and Methods:* Freshly cultured primary rat hepatocytes were treated with ascorbic acid, ALA, silymarin and their combination, both with and without the addition of APAP to evaluate their in vitro impact on cell proliferation and mitochondrial activity. In vivo study was performed on rats supplemented with the test compounds or their combination for one week followed by two toxic doses of APAP. *Results:* Selected liver function tests and oxidative stress markers including superoxide dismutase (SOD), malondialdehyde (MDA) and oxidized glutathione (GSSG) were detected. The in vivo results showed that all three pretreatment compounds and their combination prevented elevation of SOD and GSSG serum levels indicating a diminished burden of oxidative stress. Moreover, ascorbic acid, ALA and silymarin in combination reduced serum levels of liver enzymes; however, silymarin markedly maintained levels of all parameters to normal ranges. Silymarin either alone or combined with ascorbic acid and ALA protected cultured rat hepatocytes and increased cellular metabolic activity. The subjected agents were capable of significantly inhibiting the presence of oxidative stress induced by APAP toxicity and the best result for protection was seen with the use of silymarin. *Conclusions:* The measured liver function tests may suggest an augmented hepatoprotection of the combination preparation than when compared individually.

## 1. Introduction

Acetaminophen (paracetamol or N-acetyl-p-aminophenol) (APAP) is a derivative of para-aminophenol. It is widely used as a safe analgesic and antipyretic drug [1]. APAP is metabolized in the liver to excretable glucuronide and sulphate conjugates. However, high doses of APAP can cause hepatotoxicity mainly due to the formation of a highly reactive metabolite, namely N-acetyl-p-benzoquinone imine (NAPQI), that is formed by the action of hepatic enzyme cytochrome P-450 [2]. NAPQI is initially detoxified by conjugation with reduced glutathione (GSH) to form mercapturic acid and excess of oxidized GSH (GSSG). When the rate of NAPQI formation exceeds the rate of its detoxification by GSH, it starts to covalently bind to mitochondrial proteins. This covalent binding initiates hepatic cell necrosis, accompanied by oxidative stress and inflammatory responses. Inflammation reaction in the hepatocytes is often associated with over production of reactive oxygen species (ROS) that play an important role in acute liver injury [3]. Despite varied clinical presentation of APAP hepatotoxicity, *N*-acetylcysteine remains the mainstay therapy, followed by the use of liver transplantation. Herein, compounds reducing the susceptibility of patients from suffering unintentional or suicidal APAP toxicity is of interest.

Ascorbic acid, or “vitamin C”, is a widely known vitamin abundant in different kinds of fruits and vegetables and is available as an OTC supplement [4]. Ascorbic acid plays an important physiological role in cells as a reducing agent, antioxidant and free radical scavenger [5]. A strong functional inter-dependence between GSH and ascorbic acid has been reported in vivo, as it has been noted to increase GSH levels in the liver and muscle [6,7].

Alpha lipoic acid (ALA), also known as thioactic acid, is a cofactor found in a number of multi-enzyme complexes. Supplementation of ALA has been utilized in hepatic disorders, imbalance of redox status such as ischemia-reperfusion, polyneuropathy, diabetes and hypertension [8]. Moreover, ALA is involved in the synthesis of other cellular antioxidants like ascorbic acid, vitamin E and glutathione activation [9,10].

Silymarin is a flavonoid extracted from the seed of *Silybum marianum Gaetrn* (milk thistle plant) which is a member of the aster family (*Asteraceae*). *Silybin* and *Silychristin* have been identified as its other active constituents. Owing to the plant’s antioxidant role, its seeds have been used to treat a range of liver and gallbladder disorders [11].

To assess the activity of ascorbic acid, ALA and silymarin as hepatoprotectives, several markers have been chosen to be tested in the current research. Both albumin, a major protein synthesized by hepatocytes, and cholesterol that is mainly metabolized by the liver can give an insight into how well the liver is functioning. Moreover, serum alanine transaminase (ALT), aspartate transaminase (AST), alkaline phosphatase (ALP), superoxide dismutase (SOD), malondialdehyde (MDA) and oxidized glutathione (GSSH) levels can all serve as indicators of liver function and oxidative stress.

While several investigations reported ascorbic acid, ALA and silymarin’s actions on the liver independently; however, no previous research shed light on the hepatoprotective effect of their combination. Therefore, the objective of the current study is to evaluate the in vivo and in vitro hepatoprotective actions of oil-in-water emulsions of ascorbic acid, ALA and silymarin, both independently and in combination.

## 2. Materials and Methods

### 2.1. Animals

Adult male Sprague Dawley rats with an average weight of 220 ± 20 g were acclimatized at the Animal House of the University of Petra, Amman, Jordan. Rats were housed under controlled conditions such as temperature (22–24 °C), humidity (55–65%), and photoperiod cycles (12 h light/12 h dark). Rats were fasted overnight (for 18 to 22 h) with water offered ad libitum, unless otherwise stated. All experiments were performed in accordance with the University of Petra Institutional Guidelines on Animal Use, which adopts the guidelines of the Federation of European Laboratory Animal Science Association. The protocols for the animal study were revised and approved (approval number: 2-16/2/2016, date: 16 February 2016) by the Research Committee at the Faculty of Pharmacy and Medical Sciences, University of Petra (Amman, Jordan).

### 2.2. Materials and Chemicals

Ascorbic acid, alpha lipoic acid, silymarin, fetal bovine serum (FBS), streptomycin, penicillin, RPMI-1640 medium and olive oil were all purchased from Sigma Aldrich (St. Louis, MO, USA). Magnesium chloride and EDTA were obtained from Merck (Darmstadt, Germany). HBSS (Ca^2+^ and Mg^2+^ free) and HBSS (with Ca^2+^ and Mg^2+^) were obtained from Invitrogen (Carlsbad, CA, USA). Collagenase II and l-glutamine were purchased from Gibco BRL (Gaithersburg, MD, USA). Furthermore, Tween 80 and DMSO were obtained from Scharlan Chemie S.A. (Barcelona, Spain), while Tris base was purchased from Promega, (Madison, WI, USA) and TACS^®^ MTT kit was purchased from Trevigen (Helgerman Court, Gaithersburg, MD, USA). Pharmaceutical grade APAP was a kind gift from the Jordanian Pharmaceutical Manufacturing Co. PLC (JPM) Amman, Jordan. Isoflurane was supplied from Hikma pharmaceuticals, Amman, Jordan. SOD and MDA ELISA kits were purchased from MyBioSource (San Diego, CA, USA), and Glutathione ELISA kit was purchased from Sigma Aldrich (St. Louis, MO, USA).

### 2.3. Preparation of Emulsions

Tween 80 was used as the emulsifying agent while 99% olive oil was used as the constituent of the oily phase. The blank emulsion was prepared by adding tween 80 to olive oil then adding the mixture to water. ALA and Silymarin were dissolved into the oily phase whereas Ascorbic acid was added to water. Primary emulsion was prepared using 10% olive oil according to the following formula: 4 olive oil, 2 distilled water, and 1 emulsifying agent (Tween 80); then completing the volume up to 100% with distilled water [12].

### 2.4. In Vivo Experimental Protocol

A total of 48 adult male Sprague Dawley rats were weighed, randomly divided into six groups (*n* = 8), and numbered for identification. Group one and two were orally administered an oil in water (o/w) emulsion and served both as the control groups with and without APAP, respectively. Group three, four and five were orally administered with 500, 100 and 200 mg/kg of ascorbic acid [13] ALA [14] and silymarin [15], respectively. Whereas group six received a combination of the three compounds at same doses formulated as emulsions. The emulsions were administered to all groups on daily basis at the same time for eight days. Groups 2–6 were given a high dose of APAP (2800 mg/kg) at the 7th and 8th day to induce hepatotoxicity [16].

### 2.5. Blood Sampling and Testing

On the ninth day, 24 h post last toxic APAP dose, [17,18,19] all rats were euthanized by cervical dislocation, and blood samples were withdrawn by cardiac puncture, collected in sterile plain centrifugation tubes and left at room temperature for 15 min to clot completely. After that, serum was obtained by centrifugation at 6000 rpm for 10 min at room temperature and kept at −20 °C until further testing. Serum liver function biomarkers, namely ALT, ALP, AST, total albumin and cholesterol, were tested by Almahabba Laboratory (Madaba, Jordan). Oxidative stress biomarkers (SOD, MDA and GSSG) were assessed, using ELISA kits, according to the manufacturer’s recommendations.

### 2.6. Preparation of Perfusion Buffers and Culture Medium

All perfusion buffers were freshly prepared according to Al Shaker et al. (2017) using sterile technique and kept warm in a water bath at 37 °C prior use. Perfusion buffer I was prepared by adding 0.9 mM MgCl_2,_ 0.5 mM EDTA, and 0.5 mM Tris base, to 0.5 L Hank’s Balanced Salt Solution (HBSS, without Ca^2+^ and Mg^2+^) [20]. Perfusion buffer II was prepared by adding 0.5 mM Tris base to 0.5 L HBSS (with Ca^2+^ and Mg^2+^). 1000 IU of collagenase II was added to perfusion buffer II and used after 30 min of preparation. RPMI medium was prepared by the addition of 5% FBS, 100 IU/mL penicillin and 100 mg/mL of streptomycin to RPMI 1640 medium [21].

### 2.7. Surgical Protocol and Liver Perfusion

A liver perfusion system was utilized to deliver collagenase into the rat liver as described by Alshaker et al. (2017) [20]. The rat was anaesthetized by isoflurane at a concentration of 5% for induction and 2.5% for maintenance (Kent Scientific Corporation, Torrington, CT, USA). A midline longitudinal incision was made to expose the liver and the portal vein. An 18-gauge catheter (Becton Dickinson, NJ, USA) was inserted into the portal vein and infusion was initiated with perfusion buffer I pre-warmed to 37 °C at a flow rate of 10 mL/min. Once the liver blanched to a light-brown color and all lobes began to swell, circulation was switched to perfusion with buffer II including collagenase II at 25 mL/min until the liver became mushy and pale. The liver was later dissected and placed in a sterile stoppered beaker with buffer II plus collagenase II and transferred to a sterile cell culture hood. 

### 2.8. Hepatocytes Isolation and Culturing

Within the cell culture hood, and under laminar flow, liver cells were dispersed gently and aseptically using cell scrapers in a petri dish containing buffer II plus collagenase II [22]. Cell suspension was then dispensed into centrifugation tube and washed three times at 100 rpm for 3 min at 4 °C, 200 rpm for 10 min at 4 °C and finally at 200 rpm for 10 min at 15 °C. Cells were counted under light microscope using a hemocytometer, cultured in a concentration of 0.05 × 10^6^ cell/well in a 96 well plate and allowed to recover and grow in a sterile CO_2_ incubator at 37 °C prior to the day of experiment [21].

### 2.9. Cells Treatment and MTT Assay

Ascorbic acid (0.5 mM) [23] and APAP (10 mM) [24] were prepared by suspending them in RPMI medium. ALA (2 mM) and Silymarin (4 mM) were both prepared in a minimum quantity of DMSO before RPMI medium addition. Isolated rat hepatocytes were treated with the freshly prepared suspensions and incubated for 3 h. Later, APAP suspension was added to designated wells and incubated for further 48 h in a sterile CO_2_ incubator. After incubation, MTT assay was carried out to assess the mitochondrial and cellular metabolic activity, which can reflect cell viability [25].

### 2.10. Data Analysis

Data are presented as the mean ± Standard Error of the Mean (SEM). Statistical comparisons were performed using one-way ANOVA followed by Tukey’s post hoc test for multiple comparisons using SPSS statistical software version 21 (IBM corporation, Armonk, NY, USA). A *p* value less than 0.05 was considered statistically significant.

## 3. Results

### 3.1. Alteration in Serum Liver Enzyme Levels

Administration of toxic APAP doses to the rats significantly increased serum levels of ALT, AST and ALP compared to the control group Table 1. Groups treated with Ascorbic acid, ALA and Silymarin reported a significant preservation in levels of ALT and AST while only Ascorbic acid and ALA treated groups showed significantly retained levels of ALP in comparison to APAP treated group. The combination preparation significantly maintained serum levels of ALT, AST in comparison to APAP group and ALP serum level comparable to those of the control group. The effect of APAP and the tested preparations on serum cholesterol and albumin levels was insignificant (*p* > 0.05). Nevertheless, both Ascorbic acid and combination groups showed a slight effect on maintaining albumin serum levels when compared to both APAP and control groups.

### 3.2. Superoxide Dismutase and Malondialdehyde Determinations

**Figure 1:**
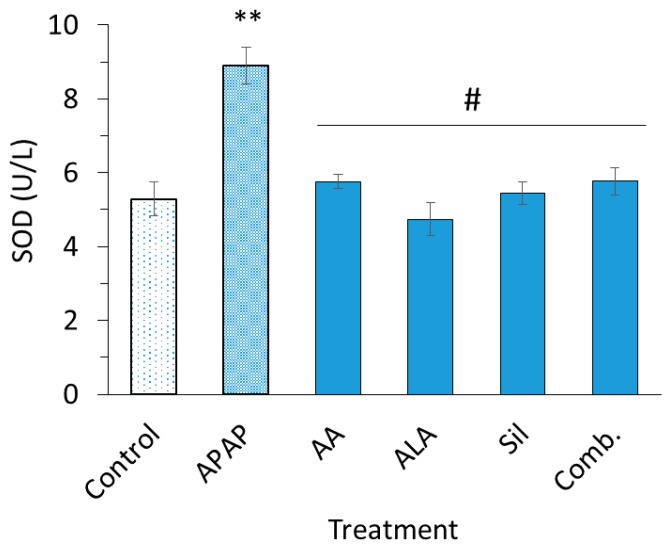
Serum superoxide dismutase (SOD) levels. SOD levels in rats intoxicated with two doses of acetaminophen (APAP) (2800 mg/kg) or intoxicated post treatment with ascorbic acid (500 mg/kg), alpha lipoic acid (ALA) (100 mg/kg), silymarin (200 mg/kg) and their combination emulsion for eight days. Each data bar represents the mean ± SEM. ** *p* < 0.05; ^#^ *p* > 0.05.

**Figure 2:**
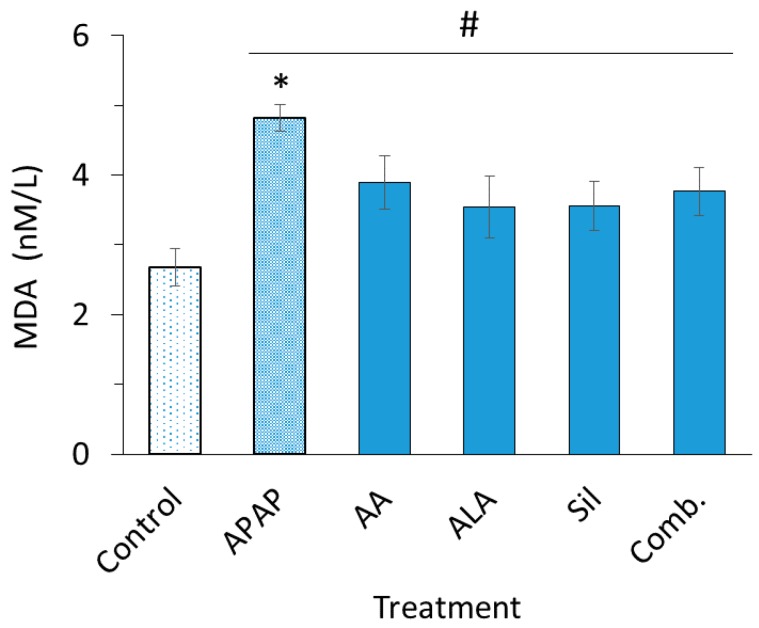
Serum malondialdehyde (MDA) levels. MDA levels in rats intoxicated with two dosed of APAP (2800 mg/kg) alone or intoxicated post treatment with ascorbic acid (500 mg/kg), ALA (100 mg/kg), silymarin (200 mg/kg) and their combination emulsion for eight days. Each data bar represents the mean ± SEM. * *p* < 0.05 compared to control group; ^#^ *p* > 0.05.

Detected serum levels of SOD and MDA in treated rats are illustrated in Figure 1; Figure 2, respectively. APAP significantly increased both serum SOD (*p* < 0.01) and MDA (*p* < 0.05) levels compared to the control group. Treating rats with ascorbic acid, ALA, silymarin and their combination preparation preserved serum levels of SOD when compared to APAP group (*p* < 0.05) and restored SOD levels to an extent equivalent to the control group. On the other hand, it was found that serum levels of MDA in rats pretreated with ascorbic acid, ALA, silymarin or their combination lowered APAP-induced MDA elevation found statistically insignificant (*p* > 0.05). 

### 3.3. Glutathione Serum Levels

**Figure 3:**
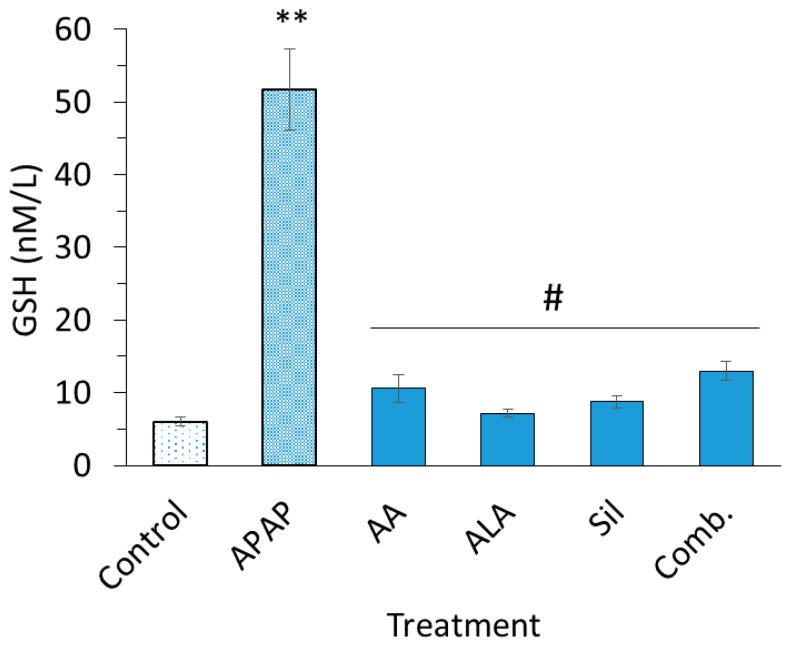
Serum glutathione (GSH) levels. Serum GSH levels of rats intoxicated with two dosed of APAP (2800 mg/kg) alone or intoxicated post treatment with ascorbic acid (500 mg/kg), ALA (100 mg/kg), silymarin (200 mg/kg) and their combination emulsion for eight days. Each data bar represents the mean ± SEM. ** *p* < 0.01; ^#^ *p* > 0.05.

Levels of GSSG were significantly increased in APAP group compared to the control group (*p* < 0.05). Pretreatment with ascorbic acid, ALA, silymarin and their combination showed a significantly attenuated APAP-induced increase in levels of GSSG, maintaining it to levels equivalent to control group levels (Figure 3).

### 3.4. In Vitro Cell Proliferation

**Figure 4:**
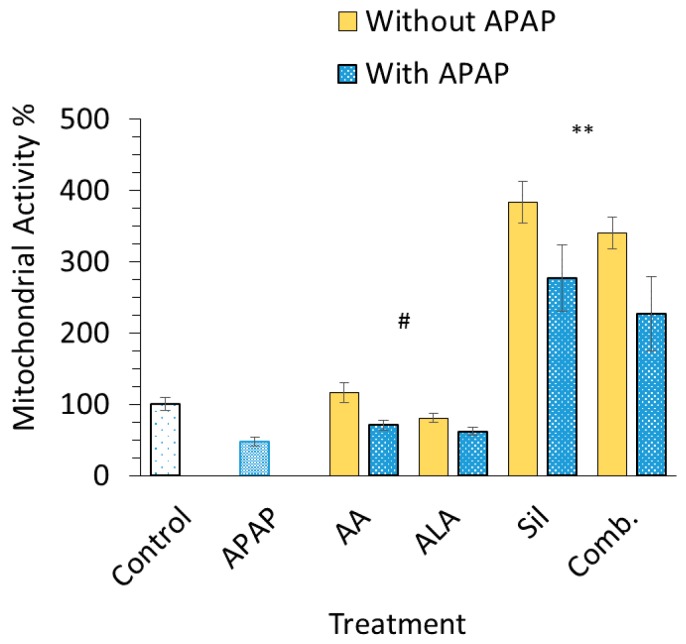
Percentage of mitochondrial activity. Percentage of mitochondrial activity of isolated rat hepatocytes incubated with and without intoxication with 10 mM APAP. The hepatocytes were pretreated with ascorbic acid, ALA, silymarin or their combination emulsion for 3 h prior the addition of APAP to induce cytotoxicity. Each data point represents the mean ± SEM (*n* = 6). ** *p* < 0.01, ^#^ *p* > 0.05 compared to control group.

In vitro testing was carried out on freshly cultured hepatocytes using MTT assay. The percentage of cell survival significantly decreased in cells incubated with APAP at a concentration of 10 mM (*p* < 0.05) compared to the control group. Silymarin and combination-treated cell groups showed a threefold increase in mitochondrial activity among other preparations with over 350% ± 48%; nonetheless, an increase in mitochondrial activity was observed when cells were pretreated with either silymarin or the combination prior to APAP (*p* < 0.05). Ascorbic acid and ALA had minor influence in terms of cell survival compared to untreated cells (Figure 4).

## 4. Discussion

The current work focused on evaluating the individual and combined hepatoprotective effects of ascorbic acid, ALA and silymarin using in vivo and in vitro models of APAP induced hepatotoxicity.

As previously demonstrated, in vivo administration of two toxic doses of APAP (2800 mg/kg) was found capable of inducing hepatic damage in rats [16]. Such dose would be expected to inflict more damage on mice due to their susceptibility to APAP-induced injury unlike rats that normally show more resistance [26,27].

Albumin levels insignificantly decreased in all groups when compared to control levels reflecting potential hepatocyte impairment in albumin synthesis due to APAP toxicity. Moreover, liver damage can cause the hepatocytes to lose their ability to metabolize cholesterol and usually results in cholesterol accumulation intracellularly, thus elevating serum cholesterol levels, as shown previously by Korkmaz et al. (2010) and Arguello et al. (2015), who reported elevation of cholesterol serum levels in liver injury [28,29]. However, the effect of ascorbic acid, ALA, silymarin and their combination in raising serum cholesterol levels was slight and statistically insignificant. 

Increased levels of AST, ALT and ALP can be indicative of cellular leakage and loss of functional integrity of hepatocyte cell membrane. This hepatocellular leakage caused these enzymes to leak out of the liver into the circulation. Serum ALT levels is considered more specific to the liver and a better parameter to evaluate the extent of liver injury [30]. In addition, elevated serum ALP levels is indicative of increased biliary pressure. Overall, significant increase in AST, ALT and ALP levels in APAP group is indicative of liver damage and cell necrosis that might be attributed to the accumulation of APAP toxic metabolite, namely NAPQI [11,31].

Ascorbic acid, ALA, silymarin and their combination were able to significantly reduce ALT and AST values in the APAP-injured animals to a level equivalent to the control group. This suggests that these compounds can prevent the effect of APAP damage and restore normal hepatocyte functions by antagonizing the toxic effect of ROS [3]. The most considerable effect in serum ALT, AST and ALP levels was seen in the group pretreated with the combination suggesting that the combined effect of ascorbic acid, ALA and dilymarin can further enhance the restoration of normal liver functions compared to treatment with either compounds alone. Enhanced effect of combined administration was also reported by Zoheir et al. (2014) and Sabiu et al. (2015), who used multiple antioxidant therapy to restore normal hepatocyte function [11,32].

Increased levels of SOD after APAP treatment is considered a biomarker of oxidative stress and injury; as reported, SOD initiates a cascade of lipid peroxidation and further generation of reactive species [33]. In the current investigation, ascorbic acid, ALA, silymarin and their combination showed significant influence in keeping SOD levels close to norm. Furthermore, the restoration of SOD levels to normal ranges through pretreatment with mentioned antioxidant chemicals presents their capacity in reducing the oxidative burden on the liver. GSSG levels were found increased upon treatment with APAP confirming the uptake of antioxidant GSH, and significantly depleting GSH through its oxidation yielding to high levels of GSSG [34]. GSSG levels in groups pretreated with ascorbic acid, ALA and dilymarin were comparable to levels of control group indicating their capability in countering ROS.

Having known that MDA is one of the end by-products of lipid peroxidation which serves as another major biomarker for oxidative stress, APAP significantly increased the levels of serum MDA which indicates the infiltration of free radicals in the tissues and lipid peroxidation. While ascorbic acid, ALA, silymarin and their combination have shown decreased levels of serum MDA, this decrease was not considered significant. Korkmaz et al. (2010) used ascorbic acid in a 50-day administration to achieve a counteracting effect against lipid peroxidation in liver-induced oxidative stress [28]. Hence, it is possible that these compounds need more time to exert their protective effect on the liver against lipid peroxidation which is translated by the levels of serum MDA.

Treating primary cultured rat hepatocytes with 10 mM APAP resulted in about 45 ± 6% reduction in cell proliferation and metabolic activity. This served as a model for evaluating the hepatoprotective actions of ascorbic acid, ALA, silymarin and their combination. An increase in the mitochondrial activity was highest in silymarin and combination treated cells and significantly higher than both APAP and control cells (Figure 4). It has been recently reported by de Oliveira et al. (2015) that Silymarin is active as an antioxidant in liver cells undergoing oxidative stress similar to that induced by APAP [35]. However, the considerable increase in the mitochondrial activity signal in cells pretreated with silymarin, assessed by MTT assay detecting reduced pyridine nucleotides, namely NADPH, that represent mitochondrial function, is accounted to the influence of silymarin on the mitochondria. Silymarin is reported to enhance NADPH levels, redox homeostasis and reduce downstream effects in toxin-induced mitochondrial dysfunction, [36,37,38] and that is resembled in Figure 4 through its positive influence on cells pretreated with silymarin prior to APAP administration. While ascorbic acid presented enhanced mitochondrial activity in cultured cells by about 16%, as described in Figure 4, such increase was not considered statistically significant. Furthermore, ALA did not noticeably alter cell survival percentages when incubated with primary cultured rat hepatocytes. Hence, it can be concluded that the combination group effect was mainly attributed to the presence of silymarin, and since the hepatoprotective actions of ascorbic acid and ALA is well documented in the literature, such low effects might be enhanced by dose adjustments or prolonged exposure time in order to obtain a noticeable effect in vitro [10]. On the other hand, ALA had positive and significant results against APAP toxicity in vivo but it did not show significant results in vitro. This might be attributed to the fact that ALA needs specific in vivo conditions to produce its metabolite, namely dihydrolipoic acid, which has been also reported to be hepatoprotective [3].

## 5. Conclusions

Pretreatment with anti-oxidative agents, namely ascorbic acid, ALA and silymarin, showed significant in vivo and in vitro influence in inhibiting the occurrence of hepatic injury induced by APAP. As presented, results showed their capacity in maintaining SOD, GSH and MDA levels to levels approximately equivalent to that of the control group. All pretreatments were capable of attenuating the insult on liver functions differently; moreover, the combination treatment showed a significant effect in containing the liver enzymes with normal ranges. Despite the significant role of Silymarin and the combination in enhancing cell viability in vitro, the capability of other agents seemed insignificant. That raises the potential additive effect of silymarin when used concomitantly with other antioxidant or hepatoprotective agents. Such protection might be attributed to the reduction of APAP-induced ROS hepatotoxicity, as evident by decreased serum levels of SOD and GSSG.

In conclusion, ascorbic acid, ALA, silymarin and their combination proved to be active as hepatoprotectants both in vivo and in vitro. Due to their different actions, the combined administration of the three agents in vitro may show superiority in enhancing antioxidative effect and hepatoprotection when compared to individual administration. However, all three compounds and their combination presented similar capability in providing hepatoprotective effects and maintaining SOD, GSH and MDA levels to within normal range. As for future implications, the current investigation suggests a possible application of the tested compound in protecting the liver of people susceptible for APAP toxicity during pain and fever management.

## Figures and Tables

**Table 1:** Selected liver function biomarkers determinations in rat serum.

Treatment (*n* = 8)	ALT ^a^	AST ^a^	ALP ^a^	Albumin ^b^	Cholesterol ^c^
Control	55 ± 6.5 **	190 ± 29 **	150 ± 58	4.6 ± 0.4 ^#^	90 ± 4.2 ^#^
APAP	3100 ± 703 **	2570 ± 241 **	449 ± 82 ^$^	3.2 ± 0.2	110 ± 8.3
Ascorbic acid + APAP	1278 ± 259	1008 ± 221	263 ± 32	2.5 ± 0.2	82 ± 8.5
ALA + APAP	807 ± 204	787 ± 191	195 ± 19	2.9 ± 0.1	102 ± 8.6
SIL + APAP	890 ± 126	916 ± 165	304 ± 57	3.1 ± 0.1	112 ± 9.3
Combination + APAP	609 ± 230	617 ± 173	148 ± 24	2.6 ± 0.1	88 ± 4.4

^a^ U/L, ^b^ g/dL, ^c^ mg/dL. ** *p* < 0.01 compared to other groups. ^$^ *p* < 0.05 compared to control. ^#^ *p* > 0.05 compared to other groups.
